# Long-term results of a phase II study with neoadjuvant docetaxel chemotherapy and complete androgen blockade in locally advanced and high-risk prostate cancer

**DOI:** 10.1186/1756-8722-7-20

**Published:** 2014-03-05

**Authors:** Mark Thalgott, Thomas Horn, Matthias M Heck, Tobias Maurer, Matthias Eiber, Margitta Retz, Michael Autenrieth, Kathleen Herkommer, Bernd J Krause, Jürgen E Gschwend, Uwe Treiber, Hubert R Kübler

**Affiliations:** 1 Department of Urology, Klinikum rechts der Isar, Technische Universität München, Ismaninger Str. 22, Munich 81675, Germany; 2 Department of Radiology Klinikum rechts der Isar, Technische Universität München, Ismaninger Str. 22, Munich 81675, Germany; 3 Department of Nuclear Medicine, Universitätsklinikum Rostock, Schillingallee 35, Rostock 18057, Germany

**Keywords:** Chemohormonal therapy, Complete androgen blockade, Docetaxel, Prostate cancer, Neoadjuvant treatment

## Abstract

**Background:**

Patients with locally advanced and high-risk prostate cancer (LAPC) are prone to experience biochemical recurrence despite radical prostatectomy (RP). We evaluated feasibility, safety and activity of a neoadjuvant chemohormonal therapy (NCHT) with 3-weekly full dose docetaxel and complete androgen blockade (CAB) in locally advanced and high-risk prostate cancer patients (LAPC) undergoing RP.

**Methods:**

Patients (n = 30) were selected by Kattans’ preoperative score and received trimestral buserelin 9,45 mg, bicalutamide 50 mg/day and 3 cycles docetaxel (75 mg/m^2^) followed by RP. Primary endpoints were biochemical (PSA) and local downstaging. Secondary endpoints included toxicity and operability assessments, pathological complete response (pCR), time to PSA progression, 5-year biochemical recurrence free survival (bRFS) and overall survival (OS).

**Results:**

Median baseline PSA was 25.8 ng/ml (2.1–293), and the predicted probability of 5-year bRFS was 10% (0–55). NCHT induced PSA-reduction was 97.3% (81.3-99.9%; p < 0.001) and post-RP 96.7% of patients were therapy responders, with undetectable PSA-values. Post- vs. pretreatment MRI indicated a median tumor volume reduction of 46.4% (−31.3-82.8; p < 0.001). A pathological downstaging was observed in 48.3%. Severe hematologic toxicities (≥CTC3) were frequent with 53.8% leucopenia, 90% neutropenia and 13.3% febrile neutropenia. RP was performed in all patients. While resectability was hindered in 26.7%, continence was achieved in 96.7%. Pathologic analyses revealed no pCR. Lymph node- and extracapsular involvement was observed in 36.7% and 56.7% with 33.3% positive surgical margins. After a median of 48.6 (19.9-87.8) months, 55.2% of therapy responders experienced PSA-recurrence. The estimated median time to PSA-progression was 38.6 months (95%CI 30.9-46.4) and 85.3 months (95%CI 39.3–131.3) for OS. The 5-year bRFS was improved to 40%, but limiting for interpretation adjuvant treatment was individualized.

**Conclusions:**

NCHT is feasible despite high hematotoxicity, with excellent functional results. Significant downstaging was observed without pCR. NCHT seems to improve the cohort adjusted 5-year bRFS, but clinical value needs further investigation in randomized trials.

## Background

Patients with locally advanced and high-risk prostate cancer (LAPC) are prone to experience biochemical recurrence despite curative-intended radical prostatectomy (RP). Predictor variables are PSA-value, clinical stage and Gleason score [1,2]. In order to improve clinical outcome, neoadjuvant regimens combined with curative treatment options are investigated [3]. In contrast to approved androgen deprivation therapies (ADT) concomitant to external beam radiation, there is no standardized systemic perioperative regimen for LAPC patients undergoing RP [3-5]. Presurgical ADT induces downstaging with reduced positive surgical margins and lymph node metastases but fails to improve survival or to induce relevant pathological complete response (pCR) rates [6,7].

In castration-resistant prostate cancer patients (CRPC), docetaxel chemotherapy (D) is standard of care due to prolonged survival in randomized phase III trials [8-11]. Subsequently, docetaxel was investigated for neoadjuvant treatment in patients with locally advanced PC. Similar to neoadjuvant ADT, preoperative docetaxel monotherapy did not improve survival and no pCR was observed, despite PSA-reduction and local downstaging [3,12-14].

Since androgen-dependent and -independent cell subpopulations may coexist, neoadjuvant strategies combining docetaxel and complete androgen blockade (CAB) were investigated, still resulting in deficient pCR rates of 3-6% [15,16]. In favor of reduced morbidity, the latter trials used weekly regimens of docetaxel, albeit 3-weekly administered docetaxel demonstrated superior clinical efficacy in comparison to weekly schedules, in CRPC patients [8,9].

The combination of CAB with a 3-weekly full dose regimen of docetaxel (75 mg/m^2^), over a period of 3 cycles (9 weeks), may exhibit superior activity to downstage tumors and improve oncological outcome. Therefore, we prospectively determined feasibility, safety and activity of this presurgical short-term combination therapy in 30 LAPC patients undergoing RP.

## Results

### Patient characteristics

The clinical patient characteristics are outlined in Table 1. Between July 2005 and February 2010, 30 patients were enrolled. Median pretreatment values were 25.8 ng/ml for PSA, 7 for biopsy Gleason score, cT3b for clinical stage and 68 years for age. According to Kattan’s nomogram, the median probability of bRFS was 10% (range 0–55) [1].

**Table 1:** Pretreatment clinical characteristics

**Clinical characteristics**	**Value**
Patients (n)	30
Age (yr.)	
Median (mean)	68 (65.9)
Range	52-76
ECOG	30 (100%)
0	
Prostate specific antigen (ng/ml)	
Median (mean)	25.8 (43.2)
Range	2.1-293.0
Gleason score at diagnosis	
6	3 (10.0%)
7	14 (46.7%)
8	6 (20.0%)
9	7 (23.3%)
Clinical stage	
T2c	2 (6.7%)
T3a	6 (20.0%)
T3b	21 (70.0%)
T4	1 (4.3%)
Kattan score	
Median (mean)	172 (171)
Range	125-200
Probability of 5-year bRFS (%)	
Median (mean)	10 (10)
Range	0-55

*Abbreviation*: bRFS, biochemical recurrence free survival.

### Neoadjuvant treatment and toxicity profile

Overall 86 cycles of docetaxel were administered and 27 (90%) patients completed all 3 cycles. Three patients (10%) discontinued NCHT; one withdrew consent after one cycle, and two interrupted due to NCHT-unrelated severe AE (lumbar disk herniation; exacerbating peripheral arterial occlusion disease (PAOD)) after two cycles of docetaxel. Bicalutamide was continued up to RP except for the patient with PAOD. Dose reductions of docetaxel were necessary in two patients (6.7%) and a treatment deferral in one patient (3.3%). Adverse events occurring in ≥10% of patients are presented in Table 2. Grade 3/4 non-hematologic toxicities were pneumonia (3.3%) and hyperglycemia (3.3%). Grade 3/4 hematologic toxicities were leucopenia in 53.8% (n = 14) and neutropenia in 90% (n = 18), in patients amenable for hematologic analyses one week after docetaxel administration. Febrile neutropenia was observed in 13.3%. Late toxicity assessments after a median follow up (FU) of 48.6 months (range 14.1-87.8) revealed one case (3.3%) of lower neuropathy (Grade 1). Second malignancies were observed in 2 cases (6.7%), one patient with bronchial carcinoma and another patient with bladder cancer, who received adjuvant radiation therapy.

**Table 2:** Adverse events, according to CTC grades, occurring in >10% of patients

**Toxicity**	**Patients**	**Grade 1**	**Grade 2**	**Grade 3**	**Grade 4**
	**n**	**n (%)**	**n (%)**	**n (%)**	**n (%)**
Alopecia	30	18 (60.0)	11 (36.7)	0	0
Fatigue and asthenia	30	14 (46.7)	14 (46.7)	0	0
Sensory neuropathy	30	9 (30.0)	4 (13.3)	0	0
Edema	30	16 (53.3)	0	0	0
Nausea and vomiting	30	10 (30.0)	0	0	0
Diarrhea	30	6 (20.0)	1 (3.3)	0	0
Constipation	30	4 (13.3)	0	0	0
Stomatitis	30	10 (33.3)	3 (10.0)	0	0
Febrile neutropenia	30	0	0	3 (10.0)	1 (3.3)
Skin rash	30	6 (20.0)	0	0	0
Arthralgia and myalgia	30	4 (13.3)	1 (3.3)	0	0
Perspiration	30	8 (26.7)	1 (3.3)	0	0
Dysgeusia	30	15 (50.0)	5 (16.7)	0	0
Nail changes	30	18 (60.0)	0	0	0
Hot flush	30	12 (40.0)	2 (6.7)	0	0
Depressive symptoms	30	8 (26.7)	1 (3.3)	0	0
Insomnia	30	8 (26.7)	0	0	0
Dyspepsia and abdominal pain	30	6 (20.0)	0	0	0
Sore throat and dysphagia	30	5 (16.7)	2 (6.7)	0	0
Chill	30	4 (13.3)	0	0	0
Increased urea	28	18 (64.3)	0	0	0
Increased potassium	30	4 (13.3)	0	0	0
Hypoproteinemia	23	4 (17.4)	0	0	0
Increased GGT	27	4 (14.8)	1 (3.7)	0	0
Increased GPT	27	4 (14.8)	0	0	0
Increased LDH	22	20 (90.9)	0	0	0
Anemia, day 21	25^#^	12 (48.0)	0	0	0
Anemia, day 7	21^#^	16 (76.2)	0	0	0
Leucopenia, day 7	26	5 (19.2)	4 (15.4)	10 (38.5)	4 (15.4)
Neutropenia, day 7	20	0	1 (5.0)	4 (20.0)	14 (70.0)

*Abbreviations*: CTC, Common Terminology Criteria for Adverse Events; GGT, Gamma-Glutamyl-Transferase; GPT, Glutamat-Pyruvat-Transaminase; LDH, Lactate Dehydrogenase; #, five patients excluded due to preexisting anemia at inclusion.

### Surgical and pathological outcome

All patients (n = 30) underwent RP with histopathological analyses after a median interval of 70 days from day 1 (range 61–143). Median removed lymph nodes were 16 (range 5–39). Surgical variables are depicted in Table 3 and pathological results in Table 4. Resectability was hindered due to periprostatic fibrosis in 23.3% and to increased vulnerability with increased diffuse bleeding in 3.3%. In case of periprostatic fibrosis the identification and dissection of the appropriate surgical layers was more difficult, when compared to not pretreated patients. Early re-interventions were required due to symptomatic lymphoceles (20%), hydronephrosis (3.3%) and bleeding (3.3%). Late events were urethral strictures (6.7%). Pathological analyses revealed extracapsular extension in 56.5% with positive surgical margins (R1) in 33.3% (n = 10) and lymph node involvement (pN1) in 36.7% of patients (n = 11).

**Table 3:** Surgical variables and complications

**Variable**	**Value**
Patients with radical prostatectomy, n	30
Surgery hindered, n (%)	
Fibrosis	7 (23.3)
Vulnerability	1 (3.3)
Surgery duration, min^#^	
Median (mean)	195 (208.7)
Range	140-333
Nerve sparing, n (%)	
Bilateral	6 (20.0)
Unilateral	3 (10.0)
Not possible	21 (70.0)
Intraoperative blood loss, ml	
Median (mean)	600 (769.2)
Range	100-2600
No. of transfused blood units, n	
Median (mean)	0 (0.9)
Range	0-5
Complications, needing intervention, n (%)^§^	
Symptomatic pelvic hematoma	1 (3.3)
Hydronephrosis	1 (3.3)
Lymphocele	6 (20.0)
Complications in ≥ 10% of cases, n (%)^§^	
Joint pain	3 (10.0)
Venous/pulmonary thromboembolism	5 (16.7)
Time to catheter removal, days	
Median (mean)	8 (13.1)
Range	7-47
Continence, no. of pads, n (%)*	
0	29 (96.7)
≥1	1 (3.3)
Erectile function (n = 26), n (%)*	
Potent (E 3–4)	4 (15.4)
Potent with erection aids (E 3–4)	3 (11.5)
Tumescence (E 1–2)	5 (19.2)
Impotence (E 0)	14 (53.9)

*Abbreviations*: ^#^skin incision to skin suture; ^§^within 4 weeks from surgery; *after a median follow up of 48.6 months (range 14.1-87.8).

**Table 4:** Pathological and oncological results in trials using presurgical docetaxel ± hormonal therapy

**Therapy**	**Docetaxel + Hormonal Therapy**								**Docetaxel – Single Treatment**		
**Author year**	**Current series**	**Narita 2012**	**Kim 2011**	**Mellado 2009**	**Sella 2008**	**Chi 2008**	**Prayer-Galetti 2007**	**Hussain 2003**	**Magi-Galuzzi 2007**	**Febbo 2005**	**Dreicer 2004**
Patients (n)	30	18	24 ^RP/Rad^	57	22	72	22	21	29	19	29
Docetaxel (D) Regime	3 cycles, q21, (75 mg/m^2^)	6 weeks, q7, (30 mg/m^2^)	3 cycles, q7 (3xD +1 week rest) (36 mg/m^2^)	3 cycles, q7 (3xD +1 week rest) (36 mg/m^2^)	4 cycles, q21 (70 mg/m^2^)	3 cycles, q7 (6xD +2 weeks rest) (35 mg/m^2^)	4 cycles, q21, (70 mg/m^2^)	6 cycles, q21 (70 mg/m^2^)	6 weeks, q7, (40 mg/m^2^)	6 months, q7, (36 mg/m^2^)	6 weeks, q7, (40 mg/m^2^)
LHRH Analog	buserelin 9.45 mg (1x3 months)	leuprorelin 11.25 mg (2x3 months)	n.d.	goserelin 10.8 mg (1x3 months)	goserelin 3.6 mg (3x1 month)	buserelin 6.6 mg (3x2 months)	triptorelin 3.75 mg (4–12 months)	n.d.	n.d.	n.d.	n.d.
Antiandrogens	bicaluta-mide 50 mg/d (9 weeks)	bicaluta-mide 81 mg/d (12 weeks)	n.d.	flutamide 750 mg/d (12 weeks)	bicaluta-mide 50 mg/d (12 weeks)	flutamide 750 mg/d // bicaluta-mide 50 mg/d (4 weeks)	n.d.	n.d.	n.d.	n.d.	n.d.
Estramustine	n.d.	1120 mg/d (6 weeks)	420 mg/d for 3d	n.d.	840 mg/d for 5d	n.d.	600 mg/m^2^ (12 weeks)	840 mg/d for 3d	n.d.	n.d.	n.d.
Therapy-weeks	9	6	12	12	12	24	16-60	18	6	24	6
pCR (%)	0	11.1	0	6	0	3.1	5	0	0	0	0
pMRD (%)	3.33	n.d.	n.d.	6	n.d.	25	31.6*	n.d.	n.d.	n.d.	7.14
iPSA, median, range (ng/ml)	25.8	25.8	22.3	9.7	21.2	10.8	41	16.1	n.d.	n.d.	12
	2.1-293	5.1-45.1	0.3-255	0.6-90.8	3.2-71.6	1.6-65.6	n.d.	2.4-175			2.5-43.3
iGl. sc. ≥7 (%)	90	87.4	100	95	n.d.	91	86	96	93	75	94
≥cT3 (%)	93.3	43.8	27	28	64	39	86	25	17.8	16	27
≥pT3 (%)	56.5	38.9	72.7	37	36.4	44	42	70	82.1	62	89
SVI (%)	53.3	11.1	45.5	n.d.	40.9	22	37	60	39.3	50	32
R0 (%)	66.7	100	63.6	64.7	72.7	73	74	70	75	n.d.	96
pN1 (%)	36.7	22.2	n.d.	3.9	18.1	6	21	10	14.3	0	14
FU, median, range (months)	48.6	18	24	35	23.6	42.7	53	13.1	49.5	26.5	23
	20-88	1-49	n.d.	23-47	12-55	26-66	30-64	9-18	23-72	4.5-40	1.5-36
Recur. Pts. (%)	55.2	22.2	55	35.1	45.4	30.0	58.0	29.0	57.0	63.2	29.0

*Abbreviations*: CSOS, cancer specific overall survival; cT3, clinical stage T3 with capsular penetration; pT3, pathological stage T3 with capsular penetration; D, docetaxel; FU, follow up; iGl. sc., initial Gleason score; iPSA, initial prostate-specific antigen; LHRH, luteinizing hormone releasing hormone; n.a., not applicable; n.d., not done; pN1, pathological lymph node involvement; pCR, pathological complete response; pMRD, pathological minimal residual disease with <5% PC in surgical specimens; *, pMRD with <10% PC in surgical specimens; q, treatment interval of 7 or 21 days; R0, negative surgical resection margin; RP, radical prostatectomy; Recur. Pts., patients with recurrent PC; RP/Rad, 12 patients treated with RP and 10 with external beam radiation; SVI, seminal vesicle invasion.

Long time functional outcome, following a median of 48.6 months (range 14.1-87.8), revealed continence in 96.7%. Before treatment 86.7% (n = 26) patients were potent. After RP 15.4% had spontaneous erections (E3-E4), 11.5% were potent with erection aids and 19.2% had tumescence sufficient for sexual activity but not for vaginal penetration (E1-E2).

### Clinical activity

**Figure 1:**
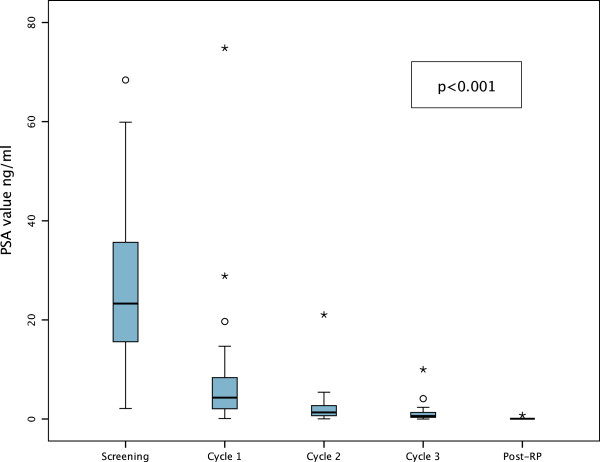
**Boxplot diagram showing prostate specific antigen (PSA)-response by treatment interval in the complete cohort of patients; extreme values (n = 3) at screening are not presented for graphing reasons; Abbreviations: **^**o**^, outliers; *, extreme outliers; RP, radical prostatectomy.

All patients (n = 30) demonstrated a PSA reduction following NCHT with a median decline of 97.3% (range 81.3-99.9%; p < 0.001) (Figure 1). Partial PSA response was observed in 100% and a complete response in 13.3%. After RP 96.7% of patients were therapy responders with undetectable PSA-values.

Downstaging of the clinical T-stage, indicated by MRI, was observed in 32% of 25 evaluable patients (cT3a to cT2 n = 3; cT3b to cT2 n = 2; cT3b to cT3a n = 2; cT4 to cT3a n = 1), without upstaging. Prostate volume was reduced in all cases with a significant median decrease of 37.1% (range 17.2-68.5%; p < 0.001). Similarly a significant median tumor volume reduction of 46.4% (range −31.3-82.8%; p < 0.001) was observed in 95.7% (n = 22) of patients harboring measurable tumors, while one progressed (4.3%). Pathological downstaging, comparing initial T-stages on MRI (n = 29) with histopathological stages revealed downstaging in 48.3% (cT3a to pT2 n = 2; cT3b to pT2 n = 10; cT3b to pT3a n = 1; cT4 to pT3b n = 1) and upstaging in 13.8% (cT2 to pT3b n = 1; cT3a to pT3b n = 3). Histopathological analyses revealed no pathological complete response (pCR), and only one (3.3%) pathological minimal residual disease (pMRD) with a tumor volume of <5%.

### Time to PSA progression and overall survival

**Figure 2:**
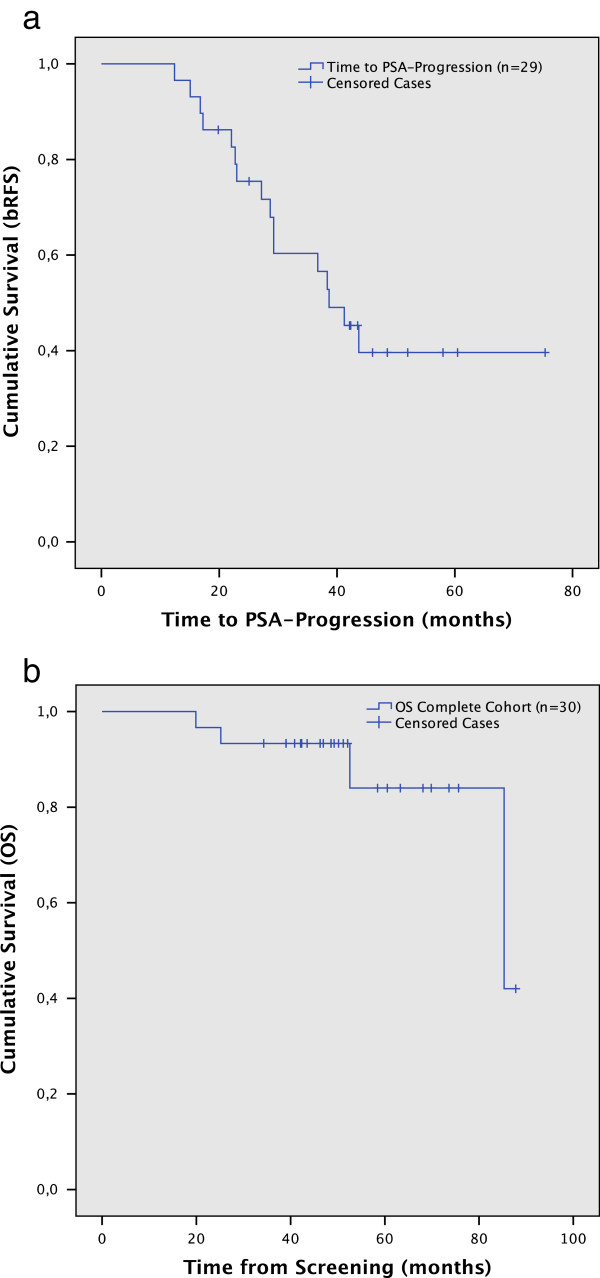
Kaplan–Meier plot for a. biochemical recurrence free survival (bRFS), in patients defined as therapy responders (n = 29). b. overall survival (OS) in the complete cohort of patients.

After a median FU of 48.6 months (range 19.9-87.8), of the 29 treatment responders 55.2% experienced a biochemical progression. The estimated median time to PSA progression was 38.6 months (95% CI 30.9-46.4) with a 5-year biochemical recurrence free survival of 40% (Figure 2a).

Adjuvant ADT was administered in 7 (23.3%) patients over a mean of 19 months (range 11–28) due to pathological R1N1 (n = 2) or R0N1 (n = 5) stages. Adjuvant radiation therapy (RT) was performed in 3 (10%) patients due to positive surgical margins without lymph node involvement (R1N0). No adjuvant RT was performed in 3 patients despite a R1N0 stage in favor a deferred RT in case of biochemical recurrence and in 4 patients with a R1N1 stage.

At the time of this analysis 26 (86.7%) patients were still alive. Two patients (6.7%) experienced a PC related death. The estimated median OS in the complete cohort was 85.3 months (95%CI 39.3–131.3) (Figure 2b).

## Discussion

We investigated a short-term neoadjuvant therapy with 3-weekly full dose docetaxel (D), combined with CAB in LAPC patients undergoing RP. Although presurgical docetaxel-based regimens were investigated earlier, FU data are mostly short-term (Table 4). In addition, investigating D + CAB, weekly docetaxel schedules were used, potentially exhibiting inferior clinical activity [8,9,15,16]. Further, a high prognostic variability was observed within and across cohorts due to study accrual following D’Amico’s criteria [2,3,15,17]. To our knowledge we are the first to present a D ± ADT treated cohort recruited according to Kattan’s nomogram, that allows for selection of equable high-risk populations [1,3,17]. Thus our cohort displays a probability of 5-year bRFS of only 10%.

With respect to non-hematologic toxicities, we observed the known profile of AE during palliative or neoadjuvant D ± ADT. The incidence of severe AE was low (7%) when compared to weekly D monotherapy (21-53%) or weekly D + CAB (21-26%) [12,13,15,16]. In contrast severe hematologic toxicities were frequent with leucopenia in 54% and neutropenia in 90%, when analyzed one week after D administration. Thus hematotoxicity was increased compared to weekly D monotherapy (0-14%) and D + CAB (0-15%) [12,13,15,16]. Our increased hematotoxicity might be caused by the 3-weekly full dose docetaxel regimen and especially by blood collection at the nadir of leucopenia.

Surgical variables are in accordance with literature. Resectability was partly hindered due to callosity as reported earlier for neoadjuvant docetaxel or CAB [7,12,13,15-19]. The rate of positive surgical margins in our series reflects the midrange of earlier published trials (Table 4). With respect to functional outcome, 97% of our patients are continent, similar to other trials reporting 89-95% continence rates [7,12,13]. Erectile function assessments revealed sexual activity in 46% of our patients, whereas 15.4% returned to baseline potency. Regrettably the majority did not try erection aids. Overall, despite explicit locally advanced stages our patients experienced excellent continence and sexual activity after RP. Comparing the excellent continence rates across neoadjuvant studies, to the results from the CaPSURE study with 63% continence and 20% potency after sole RP, the magnitude of presurgical treatment might be the amelioration of the functional outcome in patients with locally advanced stages treated with RP [20].

Several trials used a pCR rate of 10-40% as primary endpoint, but failed [7,13,15-17]. Using combinations of weekly D + CAB a pCR rate of 3-6% and pMRD rates of 6-25% were demonstrated [15,16]. In our study only one patient presented a pMRD (3%) and none a pCR. This was surprising as we used the potentially more active 3-weekly docetaxel regime. The superior results by Chi et al. might be a consequence of prolonged treatment of 24 weeks [16]. Overall neoadjuvant regimens with D ± CAB seem unable to achieve relevant pCR or pMRD rates [17]. A marginal improvement was observed by adding estramustine phosphate (EMP) to D + ADT (Table 4).

With respect to downstaging we observed a partial PSA response in 100% and a complete PSA response in 13%. The PSA-value reduction was 97%, similar to weekly D + CAB or D + EMP, but superior to docetaxel monotherapy with 64% PSA-decline and 24% partial response [12,13,15,16,21]. In addition we observed local clinical downstaging with tumor volume reduction in 96% and T-stage shift in 32%. The overall tumor volume decrease was 46%, thus being superior to D monotherapy with 26%, whereas our T-stage reduction rate is similar to ADT alone with 30% [13,22]. Finally we observed pathological downstaging in 48%, which was superior to ADT alone (15%) and comparable to D + ADT + EMP achieving 42% [7,22]. In summary, our results attained the primary endpoint with a downstaging proportion of >40% for PSA decrement, tumor volume decrease and pathological downstaging but not for clinical T-stage shift.

Neoadjuvant trials with long-term FU and relevant recurrence rates are rare. Using D + ADT + EMP a recurrence rate of 58% was observed after a FU of 53 months [7]. Similarly D monotherapy revealed a recurrence rate of 57% after a median FU of 50 months, equal to a matched control group [14]. In our trial the biochemical recurrence rate was 55% after a FU of 49 months. Thus our results are similar to the cited studies, although comparability is hindered due to different inclusion criteria. The 5-year bRFS in our cohort was increased to 40%, when compared to the predicted median of 10% [1]. However, the interpretation of our results is limited due to individualized adjuvant strategies. Further, interesting results were presented following weekly D + CAB with a recurrence rate of only 30% after a moderate briefer FU of 43 months [16]. The difference with our results might be a consequence of the longer treatment interval as well as patient selection with lower PSA-values, fewer ECE and lymph node involvement (Table 4).

Although our trial gives additional clinical information and confirms significant clinical activity of neoadjuvant D + CAB, our study has certain limitations especially with respect to bRFS. Adjuvant treatment was not uniformly defined. This is in accordance with literature, but contrasts with other trials that excluded adjuvant therapy until progression [7,12,13,15,16,23]. However due to underpowered trials, limited FU-times, variations on post-surgical management, lack of randomization and comparability, the ideal neoadjuvant approach is not yet defined [15,17]. Maybe the randomized phase III trial CALGB 90203 comparing neoadjuvant D + ADT vs. RP alone, with the primary endpoint of 3-year biochemical progression-free survival, is capable to answer the remaining questions. But to the best of our knowledge results are not on short call [24].

## Conclusions

To our knowledge we are the first to present a long time FU of a phase II trial, investigating a short-term neoadjuvant therapy with 3-weekly full dose docetaxel, combined with CAB in locally advanced and high-risk PC patients scheduled for RP. The regimen is feasible, despite a high rate of severe neutropenia and leucopenia. Significant clinical activity was observed, despite absence of pCR. Surgical variables revealed an excellent functional outcome. Finally a precariously 30% improvement of the 5-year bRFS was achieved.

## Methods

This single-center non-randomised phase II trial was conducted at the Department of Urology, Klinikum rechts der Isar, Technische Universität München. The study was approved by the institutional review board and the regulatory authorities and was performed in accordance with the ethical standards of the Declaration of Helsinki. All subjects gave written informed consent. Main eligibility criteria were adenocarcinoma of the prostate, absence of metastatic disease and a biochemical recurrence risk of >40% within 5 years, according to Kattan’s preoperative nomogram [1].

Primary objectives were downstaging effects, reflected by PSA-value decrement and local tumor reduction by clinical and pathological criteria. Secondary objectives were the pCR and pathological minimal residual disease (pMRD) rate, the toxicity profile and operability, along with time to PSA progression, 5-year biochemical recurrence free survival (bRFS) and overall survival (OS).

### Treatment

Presurgical treatment consisted of CAB comprising bicalutamide 50 mg/day p.o. starting day 1 until the day before RP with trimestral buserelin 9.45 mg s.c. on day 3 and three cycles of docetaxel chemotherapy (75 mg/m^2^) on days 8, 29 and 50 in combination with prednisolone 10 mg/day p.o. starting on day 9. Dexamethasone (8 mg p.o.) was administered 12 hours before and after docetaxel as well as 60 min before. A dose reduction of docetaxel to 60 mg/m^2^ and a treatment deferral of 21 days were allowed. All patients underwent open retropubic RP including extended bilateral pelvic lymph node dissection up to the aorta. Adjuvant radiation and hormonal therapies were individualized according to pathological results [13,23]. NCHT was aborted in case of unacceptable toxicities, progression or on patients’ request.

### Staging and clinical activity

Tumor staging included digital rectal examination, abdominal and transrectal ultrasound, chest X-ray, bone- and computed tomography scans (CT) or magnetic resonance imaging (MRI) of the pelvis. Pelvic lymph nodes were considered non-metastatic if the maximum diameter did not exceed 1.5 cm. Local clinical downstaging was assessed by MRI of the prostate with endorectal coil (1.5 T, Magnetom Avanto, Siemens, Germany) before and after NCHT. T2-weighted sequences in three planes, axial T1-weighted and a dynamic-contrast-enhanced sequence were analyzed independently by a board certified radiologist. Median prostate and tumor volumes were calculated [13,25-27]. Tumor volume was determined by measuring the maximum tumor area in the axial plane and the longest cranio-caudal diameter. In case of multiple tumor nodules, each was measured separately. Pathologic specimens were classified according to the “Union international contre le cancer” version 2002. Gleason grading is not valid following NCHT, consequently it was not determined [7,15]. The absence of PC in the histo-pathological sections was defined as pCR and tumor volumes <5% as pMRD [15]. In addition, pathologic downstaging, comparing initial stages on MRI with histopathological stages, was analyzed [7,13,22]. PSA response was assessed before RP and latest 3 months post-RP. Before RP, a PSA reduction of >50% indicated partial response and PSA-levels <0.2 ng/ml a complete response [15]. After RP, patients with PSA-values <0.07 ng/ml were deemed therapy responders. Biochemical recurrence was defined by two PSA increases >0.2 ng/ml [28].

Surgical variables were recorded implying the functional outcome. Continence was defined as the use of no pads and erectile function using the erection hardness score (E0-E4) [7,29]. Adverse events (AE) were registered according to Common Terminology Criteria (CTCAE 3.0), including dose reductions and treatment deferrals. Additional hematologic analyses were performed one week after docetaxel application.

### Statistical analysis

This trial was conducted as an intent to treat approach [30]. Sample sizes were calculated using the software N-Query version 7.0. With a sample size of 24 patients, a one-sided 97.5% confidence interval for the downstaging proportion, using the large sample normal approximation, will extend 20% from the observed proportion for an expected proportion of 40%. Analyses were performed using SPSS version 20 (SPSS Inc., Chicago, IL, USA). Changes in continuous measures over time were determined using the Wilcoxon signed-rank test and linear trend analyses using the Kruskal-Wallis test. Applying a Bonferroni correction, a p-value of <0.025 was considered statistically significant. Estimates of survival were analyzed using the Kaplan-Meier method. OS was defined as the elapsed time from screening to death and time to PSA progression as time from the first study treatment to biochemical progression [30].

## Competing interest

The authors have no actual or potential conflict of interest in relation to this article to declare. The study was supported by Sanofi-Aventis, Frankfurt, Germany.

## Authors’ contributions

MT and HRK analyzed data and wrote the manuscript, UT contributed to concept design, JG and MR directed the study and contributed to data interpretation, MT, TH, MMH, TM and MA performed the research and contributed to data collection, BJK and ME evaluated imaging datasets, KH edited the manuscript. All authors read and approved the final manuscript.
